# Damage characteristics of weak rocks with different dip angles during creep

**DOI:** 10.1038/s41598-023-34246-0

**Published:** 2023-05-09

**Authors:** Haibin Miao, Na Zhao, Lixin Meng, Yibin Zhang, Laigui Wang

**Affiliations:** 1 State Key Laboratory of Coal Mine Safety Technology, China Coal Technology and Engineering Group, Shenyang Research Institute, Shenfu Demonstration Zone, Shenyang, 113122 China; 2 College of Mechanics and Engineering, Liaoning Technical University, Fuxim, 123000 China

**Keywords:** Environmental sciences, Energy science and technology, Materials science

## Abstract

To investigate the influence of the weak layer dip angle on the creep rupture of the composite rock mass, this paper conducts a graded loading creep experiment on the composite rock mass with different dip angles using the acoustic emission method to examine the fracture evolution process. With increasing load grade, the cumulative total ring count of the rock mass shows a “U”-shaped trend, and the acoustic emission spatial positioning results show that acoustic emission events in the rock mass fracture process are primarily concentrated in the vicinity of the weak layer, while events in other areas are few and dispersed. For rock masses with weak layer dip angles of 0° and 15°, cracks occur in both soft and hard rocks, where shear cracks are dominant in soft rocks, tensile cracks are dominant in hard rocks, and finally, the rock mass mainly exhibits tensile splitting failure. For rock masses with weak layer dip angles of 30° and 45°, most of the cracks exist in the interior of the soft rock, which is dominated by shear cracks. With increasing graded loads, the shear cracks continue to develop along the direction of the weak layer, the upper rock mass keeps slipping and dislocating, and the final failure mode is mainly shear-slip failure. The damage evolution varies with the inclination angle of the weak layer, which can be divided into three stages: initial damage accumulation, damage acceleration, and damage destruction. This demonstrates the ability to predict, prevent, and control the occurrence of creep disasters in rock masses with weak layers.

## Introduction

**Figure 1:**
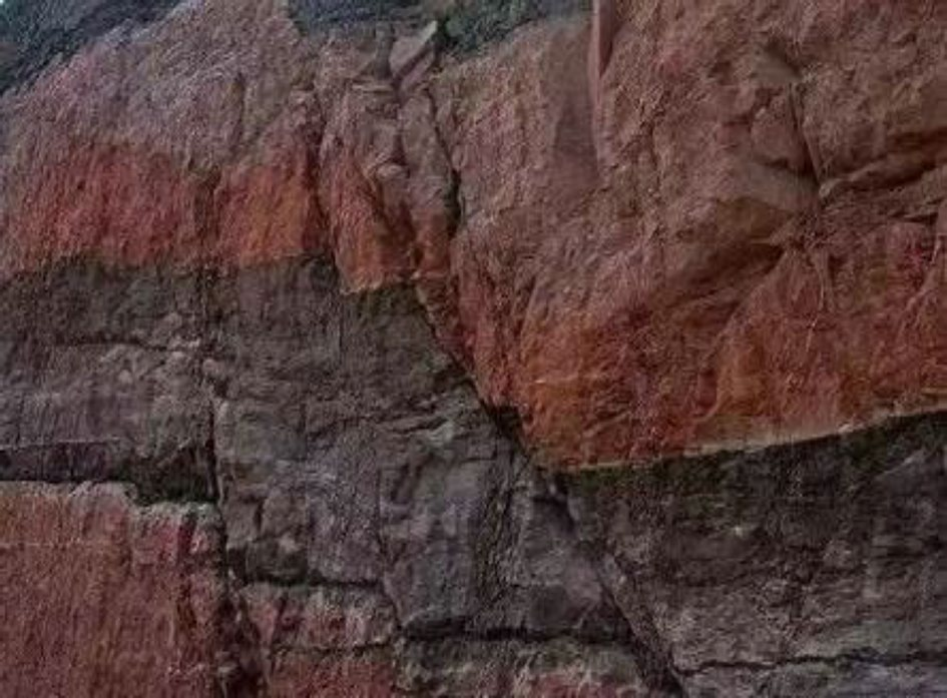
Weak-bedded rock mass.

With the rapid development of the economy, the rock mass engineering industry is growing, and more rock mass engineering projects are carried out one after another, especially in coal-measure strata, where metamorphic rocks and mudstones are widely distributed, see Fig. 1. Notably, the weak-bedded rock mass interrupts the integrity and continuity of the rock mass structure, compromises the mechanical properties of the rock mass, and increases the chances of sudden and catastrophic failure under applied stress.

The mechanical properties of the rock mass with weak layers are not only manifested in the elastic and plastic, behavior but also in the rheological behavior, which is dependent on time. Creep is one of the important rheological mechanical properties of rock masses. Destruction is the process of accumulating deformation over time under the long-term action of applied loads. Due to the existence of weak structural planes of the bedded rock mass, its mechanical properties are relatively complex, usually exhibiting significant anisotropy, and the failure mechanism is different from that of the homogeneous rock mass. To this end, many scholars have focused on elucidating the mechanical properties and failure mechanisms of composite rock masses. A brief literature review is provided below.

Xin^1^ used epoxy resin binder to combine mudstone, sandstone, and limestone into a composite rock mass, carried out the stress–strain whole process experiment of three-dimensional compression, and studied the effects of lateral pressure on mechanical properties. Changfu^2–4^ carried out uniaxial compression, triaxial compression, and direct shear experiments on various rock samples, such as mudstone, sandstone, and limestone, and three kinds of composite rock samples cemented together, to examine the strength and deformation characteristics of the layered rock mass. Yang^5^ took the slope around the Three Gorges Reservoir as a prototype, used a large-scale vibration platform to test the dynamic response and acoustic emission characteristics of the slope under earthquake action, and established the acoustic emission vibration of the sandwich slope. Bingwu^6^ carried out triaxial experiments on rock mass with weak layers and discussed the influence of interface dip on the stress–strain curve, elastic modulus, compressive strength, post-peak stress drop, and failure state of rock masses with weak layers. Additionally, the failure state of different interface inclination angles was analyzed theoretically. Li^7^ conducted uniaxial compression and triaxial compression experiments under different confining pressures on three core samples of mudstone-bearing interlayer salt rock, pure mudstone, and pure salt rock, and used the Cosserat medium expansion theory to study the shale interlayer compression. A theoretical analysis of the influence was carried out, and it was reported that the mismatch in the mechanical properties of the salt rock and the mudstone causes the mudstone to respond similarly to the application of tensile stress. Huafeng^8^ collected field samples for uniaxial compression and triaxial compression experiments and analyzed the influence of weak layer angle on the mechanical properties and failure modes. Wang^9^ considered the Hami open-pit coal mine as the research subject, determined the position of the weak interlayer in the slope by analyzing the deformation profile, regarded the weak interlayer rock mass as a complete mechanical system, established the mechanical model of the weak interlayer rock mass system, and proposed an index for evaluating the degree of failure before instability occurs. Abbas^10^ conducted research on the mechanical properties of sandstone-shale-sandstone multi-layer composite rock mass with different interlayer angles, studied the elastic response of wave speed propagation, and revealed that the elastic modulus and shear modulus of composite rock mass varies with the interlayer angle. With the increasing angle, its stiffness has a more significant effect, and the anisotropic behavior of the wave velocity in the composite rock mass is affected by the direction of the joint, not by the shale interlayer.

In addition to these fundamental experiments, numerous studies concerning the creep responses of layered rock masses have been reported. Gengyou et al.^11^ studied the creep characteristics and influence of the lamella angle on thin-layered rock structures. Yanlin et al.^12–14^ used the method of cyclic loading and unloading with graded increments to conduct creep experiments on complex ore bodies with weak inter layers and analyzed the visco-elastic–plastic deformation. To verify the correctness of the newly proposed nonlinear visco-elastic–plastic creep model, Haifei et al.^15^ conducted a triaxial compression creep experiment on sandstone under conditions of high water pressure and high confining pressure. Qiuyan^16^ conducted a series of uniaxial compression creep experiments to study the creep characteristics of argillaceous soft rocks, and analyzed the microscopic and mesoscopic changes during the creep process. Xinxi^17^ carried out triaxial compression creep experiments on argillaceous siltstone using incremental loading to study the creep characteristics and long-term strength of argillaceous siltstone under high stress.

The above studies mostly consider the mechanical properties of layered composite rock mass, but there are relatively few studies on the creep properties and fracture evolution mechanism of rock mass containing weak composites using acoustic emission equipment to observe the crack propagation with different weak layer dip angles under constant loading.

## Evolution experiment of creep characteristics of soft and weak assemblage rock mass

### Specimen preparation and scheme

**Figure 2:**
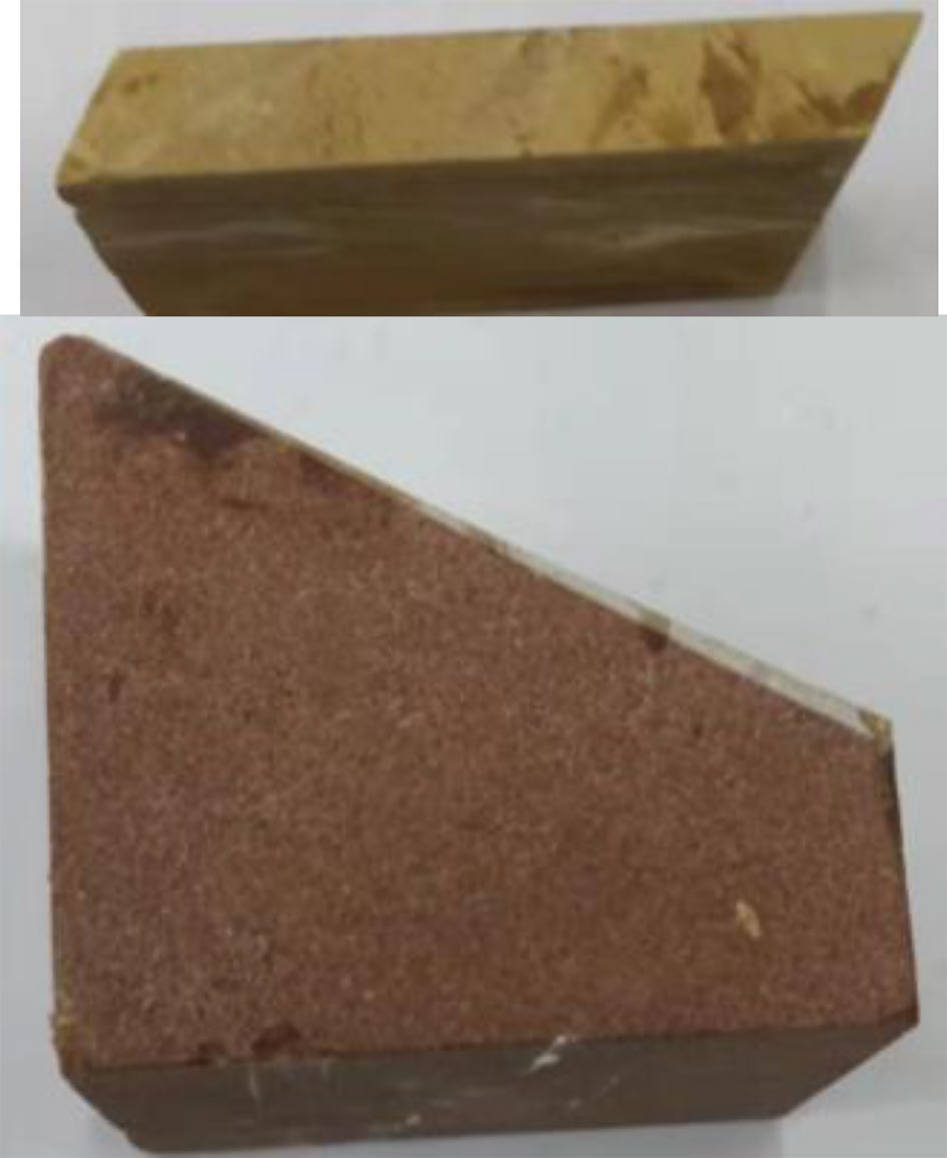
Bedrock block cut to shape.

**Figure 3:**
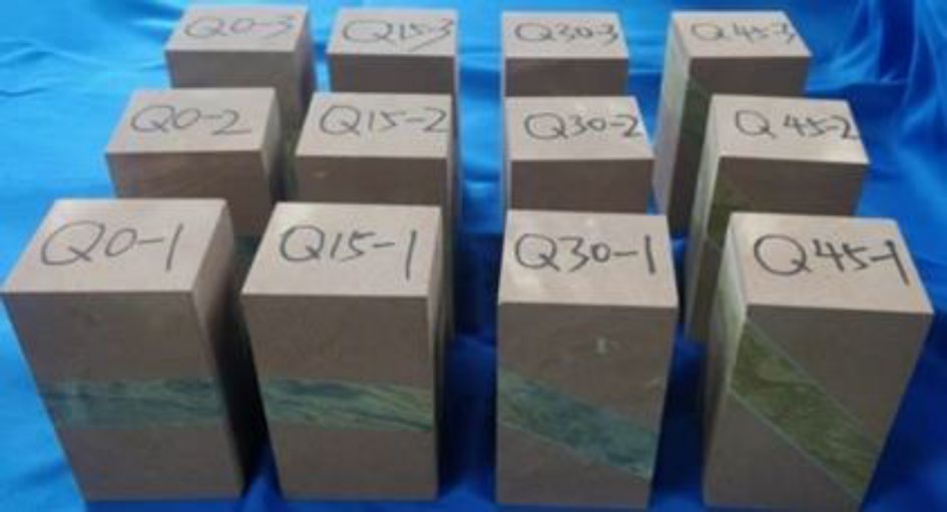
Rock mass specimen with weak bed.

According to the actual engineering geological conditions and distribution of soft rock layers, sandstone and mudstone belong to the category of sedimentary rock, which is generally composed of sand grains and weakly consolidated clay deposited on the riverbed after long-term water erosion, and the inter-layers generally form over hundreds of years of accumulation. Rock mass, due to the low strength introduced by defects of the inter-layers, is susceptible to disintegration when encountering water and exhibits poor cementing ability, which is prone to causing various geological disasters. In this test, sandstone and mudstone are selected as hard rock and soft rock respectively, and cut and polished according to the requirements of the test specification to make several bedrock blocks. As shown in Fig. 2, the parallelism and smoothness of the end face of the bedrock meet the standards of the International Society for Rock Mechanics (ISRM), ensuring that the end face error is ± 0.1 cm, keeping the surface of the bedrock clean and tidy, placing them in the mold in turn, and evenly applying marble glue at the interface, During the bonding process, it is necessary to ensure that the verticality of the rock mass meets the requirements of the test specification, and the size of the rock mass is 50 mm × 50 mm × 100 mm standard rectangular test piece, as shown in Fig. 3,The angles are divided into four groups, namely 0°, 15°, 30°, and 45°, with three samples in each group.

### Experimental design

**Figure 4:**
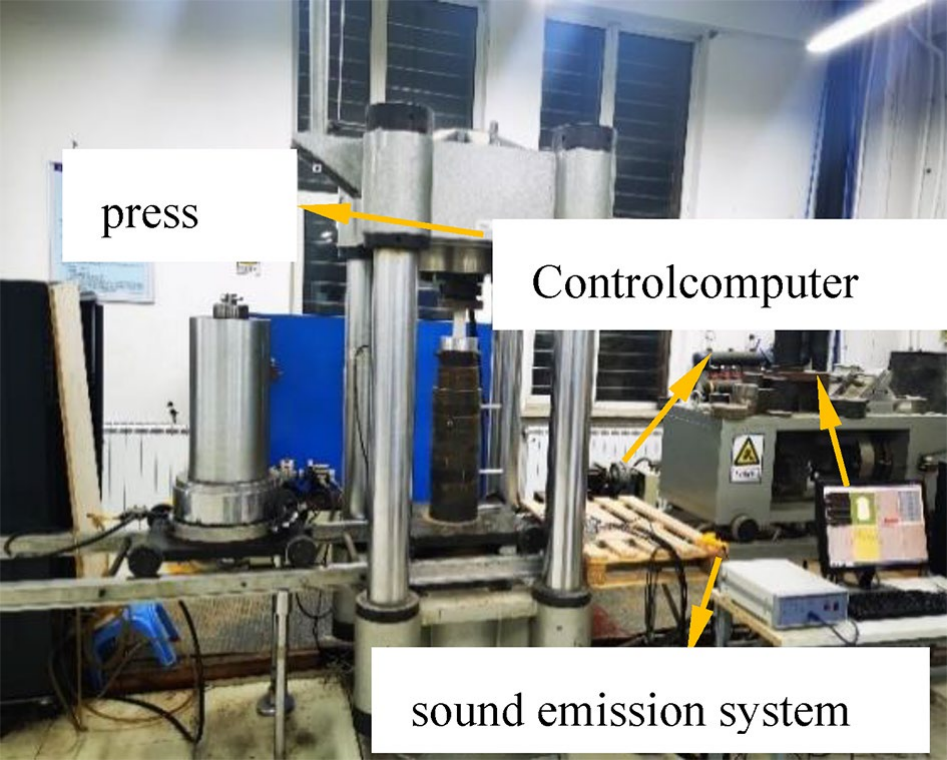
Creep loading system.

The experimental setup consists of a loading system and an acoustic emission detection system, as shown in Fig. 4. The experimental loading system uses aYAW-2000 microcomputer-controlled electro-hydraulic servo pressure testing machine. During the creep experiment, a constant load is applied using staged loading. The loading rate is 0.02 MPa/s. The first stage load is the same for each working condition. The rock mass corresponds to 35% to 50% of the uniaxial compressive strength, and the load increment of staged loading is 0.5 MPa for multistage loading creep experiments. When the creep curve tends to be stable or the rate of the stable creep stage is zero, the next stage of loading is applied until the creep test fails. The experimental machine is used to record the axial deformation during the creep process, and the acoustic emission system is used to monitor the real-time response. For the acoustic emission signal inside the body, it is necessary to pay close attention to the experimental process and fine-tune the loading time.

**Figure 5:**
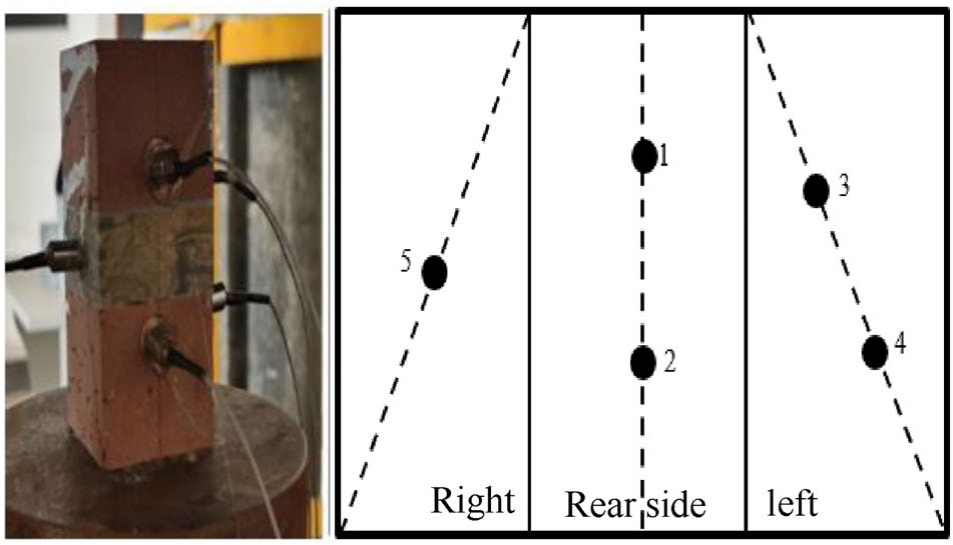
Distribution of charge sensors.

The acoustic emission monitoring system uses the PCI-2 type 8-channel acoustic emission test and analysis system produced by American Acoustic Physics Corporation. The system is mainly composed of computers, probes, preamplifiers, converters, acoustic emission signal lines, and other equipment. The acoustic emission probe signal is RS-54A, the diameter is 8 mm, the amplifier models are 20/40/60 dB, and three different gears are provided: 20, 40, and 60 dB.Its function is mainly to amplify the probe to capture and transmit signals to the computer for analysis and processing. This type of amplifier has the advantages of small size, low noise, and impact resistance. The monitoring threshold of the system is 36 dB, the resonance frequency is 140 kHz, the impact definition time is 50 µs, and the sampling time interval is 0.1 s. It mainly monitors the number of events, energy count, ringing count, and parameters such as source feature points. During the experiment, to construct the three-dimensional acoustic emission spatial distribution, five acoustic emission probes were used on the left, right, and rear sides of the sample. The probe positions are shown in Fig. 5.

## Creep test curve and acoustic emission ringing count analysis

**Figure 6:**
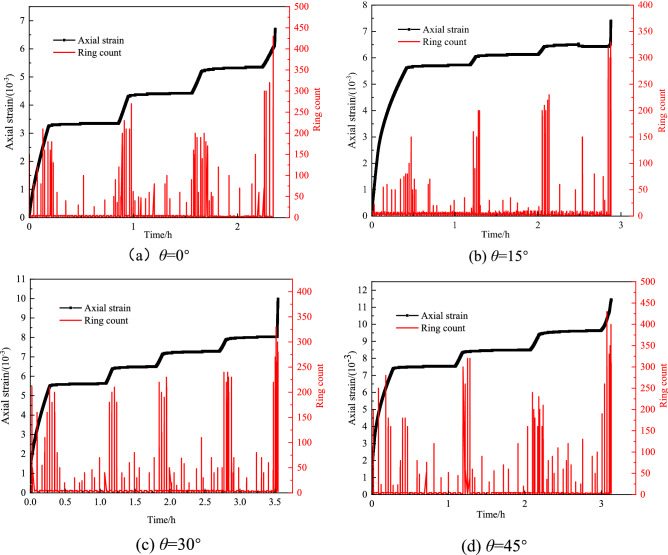
Relation curve between ring count, axial strain, and time of rock with different dip angles of weak layers in creep experiment.

The graded loading load values during the creep experiment are shown in Table 1. The acoustic emission ringing count signal inside the rock mass is shown in Fig. 6.

**Table 1:** Proposed values of loads at all levels in graded loading creep tests.

Weak layer dip *θ*/(°)	load series/MPa			
	Level 1	Level 2	Level 3	Level 4
0	2	2.5	3	–
15	1.5	2	2.5	–
30	1.5	2	2.5	3
45	1	1.5	2	-

From Fig. 6 and Table 2,when the load increases from 0 to the first-level load, the acoustic emission signals released by each group of rock masses are dense, the corresponding cumulative ringing count is higher, and the ringing count increases with the increasing inclination of the weak layer. When the first-level load is stable, the acoustic emission signal released by the rock mass gradually decreases, and the cumulative ringing count gradually decreases. When the second-level load increases, the cumulative ringing count increases again. After the second-level load is stabilized, the cumulative ringing count of each group of rock masses becomes calmer. Since the incremental load changes are small during the second-level rising load period, the cumulative ringing count is significantly lower than that of the first-level load period. The ringing counts for the creep stage under the second-level load increase slightly compared to the first-level creep stage. Under the action of the last level of load, the cumulative ringing counts in the rising load stage have increased compared with the previous two stages. In the accelerated creep stage, the total accumulated ringing counts of the samples under various loads show a trend of first decreasing and then increasing with the increasing load. The change in the dip angle of the weak layer produces a sudden change.

**Table 2:** Cumulative ring count of rock for each weak layer dip angle and stress level in creep test.

Weak dip Angle	Cumulative ringing count/10^3^			
	Level 1	Level 2	Level 3	Level 4
*θ* = 0°				
Load rising period	13.65	8.48	9.42	–
Stabilization period	5.76	6.55	25.47	–
*θ* = 15°				
Load rising period	14.26	10.43	11.88	–
Stabilization period	2.26	3.57	20.62	–
*θ* = 30°				
Load rising period	15.59	10.36	11.21	13.46
Stabilization period	6.74	7.52	8.41	19.34
*θ* = 45°				
Load rising period	16.47	13.22	14.46	–
Stabilization period	5.54	6.28	18.57	–

## Distribution characteristics of acoustic emission sources

**Figure 7:**
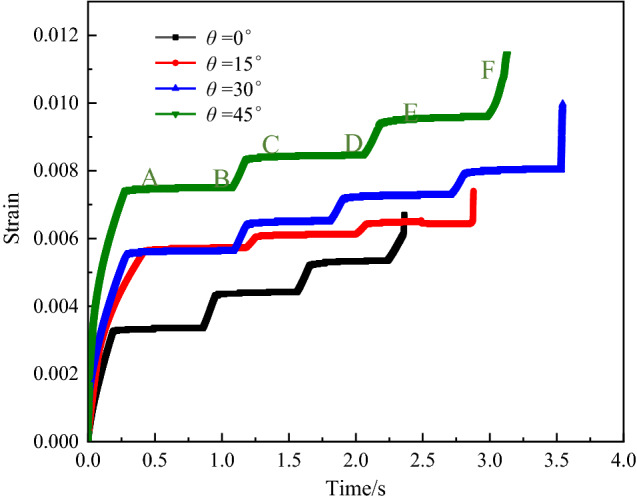
Creep curves of the rock sample containing weak layers with different dip angles.

**Figure 8:**
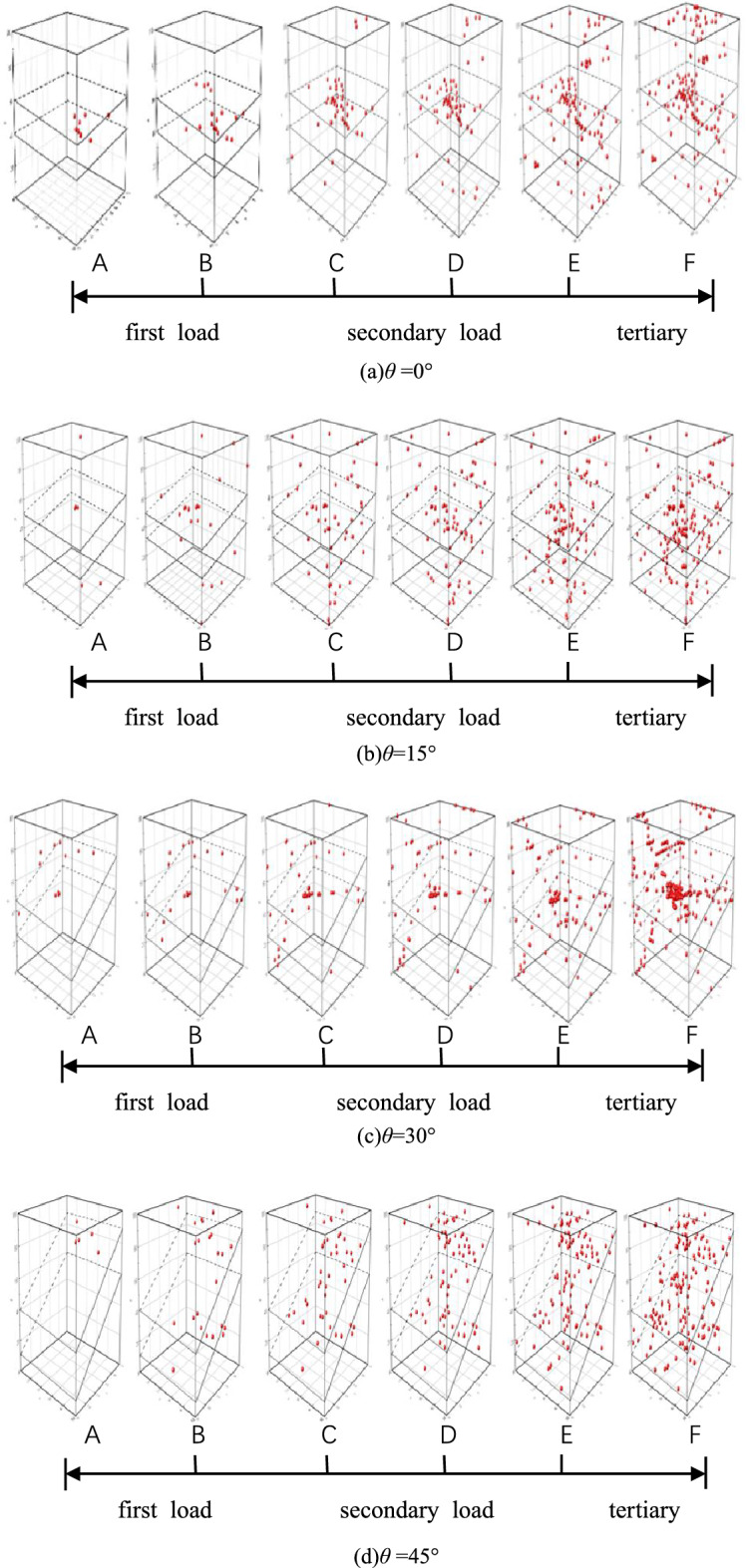
Focal distribution and evolution of rocks with different dip angles of weak layers during graded loading creep.

The creep curves of all load levels under each inclination angle are shown in Fig. 7. To facilitate the analysis, the source distributions of the various load levels under various stress levels after reaching stability are selected, as shown in Fig. 8A,C and E. The source distribution diagrams in Fig. 8B and D represent the later end of each stage, and the source distributions in Fig. 8F denote the late stage of accelerated creep, and the specific positions of each surface of the soft rock are specified in the diagrams.

From Fig. 8, when the inclination angle of the weak layer is 0° and the first-level load is stable, there is a small amount of hypocenter in the middle of the right side of the soft rock. When the second-level load is applied, the hypocenter increases slightly. At this time, a hypocenter is generated at the top of the hard rock. After the load is stabilized, the acceleration of the hypocenter gradually decreases. Only a small amount of hypocenter is generated in the hard rock on the lower side. When the load exceeds 80% of the compressive strength, the seismic source on the right surface of the soft rock and the top of the hard rock on the upper side increases significantly. After entering the acceleration stage, the seismic source develops rapidly toward the left side of the soft rock, and then the rock mass undergoes creep instability.

When the inclination angle of the weak layer is 15° and the first-level load increases to a stable level, there are hypocenters near the upper side of the soft rock and the top and bottom of the hard rock. At this time, the number of hypocenters is very small. After entering the creep stage, the long-term constant load acts. In the middle of the soft rock, there are a small number of hypocenters dispersed in the middle of the soft rock, and the hypocenters in the soft rock are more than those in the hard rock. There are also a small number of sources at the edge. After entering the creep stage, the number of sources does not change significantly. At this time, the acoustic emission signal is relatively quiet. When the third-order load is applied, the hypocenters increase significantly at the lower side of the soft rock and the lower interface. A few hypocenters are generated, and the acoustic emission signal in the creep stage also becomes very active. After entering the accelerated creep stage, the number of hypocenters increases to the maximum value, the rock mass is crushed as a whole, and finally, tensile splitting failure and shearing occur. cut damage.

When the inclination angle of the weak layer is 30°, the first-level load is applied until the stable stage and through the entire creep stage. In this case, there is no seismic source inside the soft rock and hard rock, and then the load is increased. After the load remains unchanged, the upper interface shows trace sources distributed around the soft rock and the soft rock. After entering the creep stage, the source almost did not increase. After the third-level load is applied, the source increases significantly at the interface, and part of the source occurs in the lower left corner of the hard rock on the lower side, which enters the creep stage. At the same stage, the same number of sources remains constant. When the fourth-level load is applied, the load reaches 85% of the compressive strength. The source on the right side of the soft rock increases significantly, and the accumulation of sources on the upper interface becomes more clear. After severe damage, the bearing capacity of the rock mass gradually decreases. After entering the stage of accelerated creep, a large number of hypocenters are generated and develop toward the soft rock. At this time, a "seismic source group" has been formed in the soft rock, and the rock mass eventually breaks down.

When the inclination angle of the weak layer is 45°, after stability is reached during the primary loading, a trace source is generated at the top of the upper side of the soft rock. The hypocenter is generated, and the load continues to increase. The number of hypocenters increases in the upper middle and lower middle of the soft rock. There is a trace hypocenter at the top of the hard rock. After entering the creep stage, the hypocenter increases slightly. The number of hypocenters in the middle of the rock is significantly increased, and the hypocenters are evenly distributed in the soft rock, while the hypocenters in the hard rock are extremely small.

Not only does the number of sources change with the load increase, for a given load level, the number of sources also increase with increasing weak dip angle; the greater the dip angle, the more the hypocenters tend to be distributed near soft rock and the greater the rock mass damage. It can be seen that the greater the weak dip angle, the more prone to rock mass instability failure. It can be seen that the larger the dip angle of the weak layer is, the more severe the rock mass damage is, and the more likely the instability failure will occur.

## Creep failure modes

**Figure 9:**
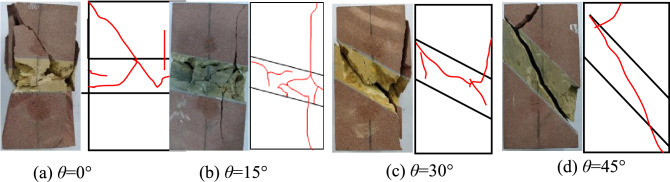
Final creep failure mode of rock mass with different weak layers and dip angles.

From Fig. 9, the failure modes of the four groups of rock samples with different dip angles of weak layers can be divided into split tensile failure and shear-slip failure along the weak interlayer. The cracks generated when the rock mass shears along the maximum shear stress are shear cracks, and the cracks generated when the rock mass shears are tensile cracks. When the dip angle of the weak layer is 0°, the surface cracks appear around the soft rock and hard rock, and the rock blocks peel off successively, and finally, the rock mass experiences tensile splitting failure. When the inclination angle of the weak layer is 15°, the vertical crack penetrates the soft-hard interface and connects with the main crack in the soft rock, and finally, the rock mass undergoes tensile splitting failure. When the inclination angle of the weak layer is 30°, due to the expansion and penetration of the surface cracks on the left side of the rock mass, the exfoliation of large blocks occurs, and a macroscopic fracture surface forms in the middle of the soft rock, and the sample slips along the macroscopic fracture surface. Eventually, the rock mass undergoes shear-slip failure. When the inclination angle of the weak layer is 45°, the cracks on both sides of the crack are connected, extend to the upper interface on the left side of the weak layer, penetrate the interface, and extend to the top of the hard rock, causing large pieces of hard rock and soft rock to fall. Macroscopic shear-slip cracks are generated, which cause shear-slip instability and failure of the rock mass.

## Analysis of acoustic emission damage characteristics of creep failure

Acoustic emission can elucidate the internal damage to the rock that evolves during the failure process. To intuitively obtain the internal damage of the rock during the loading process, the cumulative ringing count of acoustic emissions is selected as the variable to characterize the rock damage.

Kachanov^18^ defined the damage variable D as:

1 $$ D = \frac{{A_{{\text{d}}} }}{A} $$

In the formula, D represents the damage variable, A is the effective cross-sectional area of the initial state, and A_d_ is the effective cross-sectional area of the rock sample where damage occurs.

The acoustic emission ringing count per unit area when the rock is damaged is:

2 $$ N_{{\text{c}}} = \frac{{N_{{\text{w}}} }}{A} $$

In the formula, *N*_*c*_ is the cumulative ringing count when the rock sample is damaged per unit area, and *N*_*w*_ is the cumulative ringing count after the rock sample is completely damaged.

When the damaged area is any *A*_*t*_, *N*_*t*_ can be expressed as:

3 $$ N_{{\text{t}}} = \frac{{N_{{\text{w}}} }}{A}A_{{\text{t}}} $$

Combining Eqs. (1) and (3), the damage variable D can be expressed as:

4 $$ D = \frac{{N_{{\text{t}}} }}{{N_{{\text{w}}} }} $$

Based on the incomplete damage of damage, the damage variable can be corrected. Referring to the research of Liu Baoxian^19–21^ and other scholars, the critical value of damage after correction can be taken as:

5 $$ D_{{\text{t}}} = 1 - \frac{{\partial {\text{p}}}}{{\partial {\text{c}}}} $$

In the formula, *D*_*t*_is the critical damage value, *∂p* is the rock peak strength, and *∂c* is the rock residual strength. The modified damage variable can be obtained as:

6 $$ D = \left( {1 - \frac{{\partial {\text{p}}}}{{\partial {\text{c}}}}} \right)\frac{{N_{{\text{t}}} }}{{N_{{\text{w}}} }} $$

According to the equivalent strain and elastic mechanics theory, the rock damage model under acoustic emission is:

7 $$ \sigma = E\varepsilon \left( {1 - D} \right) = E\varepsilon \left[ {1 - \left( {1 - \frac{{\partial {\text{p}}}}{{\partial {\text{c}}}}} \right)\frac{{N_{{\text{t}}} }}{{N_{{\text{w}}} }}} \right] $$

**Figure 10:**
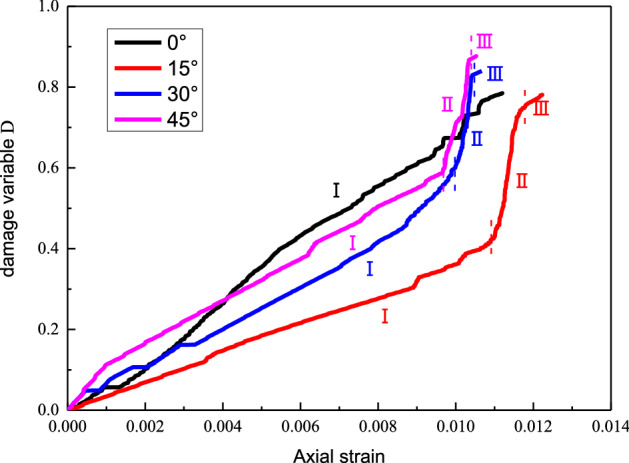
Damage evolution curves of rocks containing weak layers with different dip angles.

Sort out and calculate the cumulative ring count, the change of ring count at any time and the rock physical and mechanical parameters in the acoustic emission test results, and then substitute the results into formula (7) and draw the rock damage evolution curve of different weak layer dip angles through origin, as shown in Fig. 10.

From Fig. 10, the damage evolution curves of the rock mass vary with the dip angle of the weak layer, but it can be divided into three stages: damage accumulation, damage acceleration, and damage destruction. When the inclination angle of the weak layer is 0°, the initial damage variable of the rock is small, and the rock is gradually compacted during the loading process. When the layer dip angle is 15°, the initial damage variable is the smallest. With the gradual accumulation of damage, the rock enters the accelerated damage stage, and finally, damage occurs. When the weak layer dip angle is 30°, the initial damage variable develops flexibly with the rock compaction. With the continuous development of creep, the damage gradually accumulates, and it enters the accelerated damage stage earlier than that at 15°, and finally reaches the peak damage value. When the inclination angle of the weak layer is 45°, the initial damage variable value is the largest, and the rock is completely compacted. After that, it enters the damage accumulation stage. With the continuous increase of stress, the rock enters the failure stage, and the damage amount reaches a maximum. Ultimately, the inclination angle of the weak layer has different effects on the various stages of the rock mass.

## Conclusion

In this paper, graded loading creep experiments are carried out on the weak composite rock mass with different weak layer dip angles. An acoustic emission system is used to monitor the real-time acoustic emission signals inside the rock mass, and the graded loading creep curve, failure mode, and acoustic emission characteristics were comprehensively analyzed (ringing count and source evolution characteristics).The following conclusions are obtained:

1. With the increase of the graded load, new cracks appear inside the sample and continue to expand, connect, and penetrate, and the damage to the sample accumulates with the increased loading time. The greater the inclination of the weak layer, the greater the creep deformation of the rock mass. The cumulative ringing counts of rock masses with weak layers under various loads show a trend of first decreasing and then increasing with the increase of the load level, showing a U-shaped trend.
2. The acoustic emission spatial positioning results indicate the initial crack position inside the sample, the damage status of the rock mass, and the crack propagation under different loading stages. The temporal and spatial evolution of the source shows relatively obvious stages. The acoustic emission events in the fracture process of the composite rock mass are mainly concentrated near the soft rock, and the acoustic emission events in other areas are less frequent and more dispersed. The damage and fracture of soft rock effectively determine the overall deformation and failure of the composite rock mass.
3. The final creep failure mode of the rock mass is affected significantly by the weak layer dip angle. For the rock masses with the weak layer dip angles of 0° and 15°, cracks occur in both the soft and hard rocks, and the rock mass exhibits tensile splitting. In the rock masses with weak layer dip angles of 30° and 45°, most of the cracks exist in the soft rock, with a majority of shear cracks. With the increase of the graded load, the shear cracks continue to develop along the direction of the soft rock, the upper rock mass keeps slipping and dislocating, and the final failure mode is mainly shear-slip failure.
4. The damage evolution curves of the rocks vary with the inclination of the weak layer, but they can be divided into three stages: damage accumulation, damage acceleration, and damage destruction. The larger the inclination angle of the weak layer of the rock mass, the more accumulative ringing counts are measured, and the larger the damage variable value.

## Data Availability

The data used to support the findings of this study are available from the corresponding author upon request.

## References

[1] Xin L (1987). Study on stress-strain characteristics of composite rock mass under three-dimensional compression. J. China Acad. Min. Technol..

[2] Changfu X, Xin L, Gang W, Xiande Q (1988). Influence of interlayer bond strength on compressive mechanical properties of composite rock mass. J. Chongqing Univ. Nat. Sci. Edit..

[3] Changfu X, Xiande Q (1983). Discussion on strength and deformation characteristics of composite rock mass under uniaxial and triaxial compressive stress. J. Chongqing Univ. Nat. Sci. Edit..

[4] Changfu X, Congxin C, Gang W, Xiande Q (1987). Hypothesis of asymptotically coordinated shear failure of composite rock mass and its strength equation. J. Chongqing Univ. Nat. Sci. Edit..

[5] Yang X, Dong J, Cai W (2021). Seismic dynamic responses and acoustic emission criteria of a horizontal soft-hard interbedded slope. Arab. J. Geosci..

[6] Bingwu W, Yinping L, Chunhe Y (2015). Influence of interface dip angle on mechanical properties of composite layered physical model materials. Rock Soil Mech..

[7] Yinping L, Jiang L, Chunhe Y (2006). Analysis of the influence of mudstone interlayers on the deformation and damage characteristics of salt rock. Chin. J. Rock Mech. Eng..

[8] Huafeng D, Wei W, Jianlin L (2018). Experimental study on anisotropic mechanical properties of layered sandstone. Chin. J. Rock Mech. Eng..

[9] Wang T, Zhao H, Liu Y (2020). Formation mechanism and control measures of sliding surface about bedding slope containing weak interlayer. KSCE J. Civ. Eng..

[10] Abbas HA, Mohamed Z, Mohd-Nordin MM (2022). Characterization of the body wave anisotropy of an interbedded sandstone-shale at multi orientations and interlayer ratios. Geotech. Geol. Eng..

[11] Gengyou H, Sijing W, Xiaoping Z (2010). Research on creep characteristics of thin-layered rock mass under hierarchical loading. Chin. J. Rock Mech. Eng..

[12] Yanlin Z, Ping C, Yuanjiang C (2008). Rheological experiment and model of jointed soft rock under graded loading and unloading. Chin. J. Coal.

[13] Yanlin Z, Jinzhou T, Chengcheng F (2016). Rheological experiment and creep damage model of viscoelastic-plastic strain separation of rock mass. Chin. J. Rock Mech. Eng..

[14] Zhao YL, Zhang LY, Wang WJ (2017). The mechanical properties of mudstone at high temperatures-an experimental study. Rock Mech. Rock Eng..

[15] Haifei J, Dongyan L, Baoyun Z, Jie S (2014). Experimental study on triaxial creep properties of sandstone under high stress and high water pressure. Exp. Mech..

[16] Qiuyan F, Keqing Y, Weiming W (2010). Research on creep mechanism of argillaceous soft rock. Chin. J. Rock Mech. Eng..

[17] Xinxi L, Shengnan L, Yanming Z (2020). Creep characteristics and long-term strength of high stress argillaceous siltstone. Chin. J. Rock Mech. Eng..

[18] Katchanov LM (1958). Time of the rupture process under creep conditions. Izk. Akad. Nauk.

[19] Baoxian L, Jinglin H, Zeyun W (2009). Study on damage evolution and acoustic emission characteristics of uniaxial compression coal and rock. Chin. J. Rock Mech. Eng..

[20] Zhiwei Z, Jianfeng L, Hang Z (2016). Discussion on acoustic emission characteristics and damage evolution of uniaxial compression salt rocks. J. Yangtze Acad. Sci..

[21] Yongjie Y, Dechao W, Mingfu G (2014). Research on rock damage characteristics based on triaxial compression acoustic emission test. Chin. J. Rock Mech. Eng..

